# Spread of a Novel Indian Ocean Lineage Carrying E1-K211E/E2-V264A of Chikungunya Virus East/Central/South African Genotype across the Indian Subcontinent, Southeast Asia, and Eastern Africa

**DOI:** 10.3390/microorganisms10020354

**Published:** 2022-02-03

**Authors:** Juthamas Phadungsombat, Hisham A. Imad, Emi E. Nakayama, Pornsawan Leaungwutiwong, Pongrama Ramasoota, Wang Nguitragool, Wasin Matsee, Watcharapong Piyaphanee, Tatsuo Shioda

**Affiliations:** 1 Mahidol-Osaka Center for Infectious Diseases (MOCID), Faculty of Tropical Medicine, Mahidol University, Bangkok 10400, Thailand; juthamas@biken.osaka-u.ac.jp; 2 Center for Infectious Disease Education and Research, Department of Viral Infections, Research Institute for Microbial Diseases (RIMD), Osaka University, Osaka 565-0871, Japan; hishamahmed.ima@mahidol.ac.th (H.A.I.); emien@biken.osaka-u.ac.jp (E.E.N.); 3 Mahidol Vivax Research Unit, Faculty of Tropical Medicine, Mahidol University, Bangkok 10400, Thailand; wang.ngu@mahidol.edu; 4 Department of Microbiology and Immunology, Faculty of Tropical Medicine, Mahidol University, Bangkok 10400, Thailand; pornsawan.lea@mahidol.ac.th; 5 Center of Excellence for Antibody Research (CEAR), Department of Social and Environmental Medicine, Faculty of Tropical Medicine, Mahidol University, Bangkok 10400, Thailand; pongrama.ram@mahidol.ac.th; 6 Department of Molecular Tropical Medicine and Genetics, Faculty of Tropical Medicine, Mahidol University, Bangkok 10400, Thailand; 7 Hospital for Tropical Diseases, Faculty of Tropical Medicine, Mahidol University, Bangkok 10400, Thailand; wasin.mat@mahidol.edu (W.M.); watcharapong.piy@mahidol.ac.th (W.P.); 8 Department of Clinical Tropical Medicine, Faculty of Tropical Medicine, Mahidol University, Bangkok 10400, Thailand

**Keywords:** chikungunya virus, East/Central/South African (ECSA) genotype, Indian Ocean Lineage, outbreak, molecular clock analysis, mutation, mosquito

## Abstract

The Indian Ocean Lineage (IOL) of the chikungunya virus (CHIKV) East/Central/South African (ECSA) genotype, which originated in Kenya, spread to the Indian ocean and the Indian subcontinent, and then expanded through Southeast Asia in the previous decade. It carried an adaptive mutation E1-A226V, which enhances CHIKV replication in *Aedes albopictus*. However, the IOL CHIKV of the most recent outbreaks during 2016–2020 in India, Pakistan, Bangladesh, the Maldives, Myanmar, Thailand, and Kenya lacked E1-A226V but carried E1-K211E and E2-V264A. Recent CHIKV genome sequences of the Maldives and Thailand were determined, and their phylogenetic relationships were further investigated together with IOL sequences reported in 2004–2020 in the database. The results showed that the ancestral IOLs diverged to a sub-lineage E1-K211E/E2-V264A, probably in India around 2008, and caused sporadic outbreaks in India during 2010–2015 and in Kenya in 2016. The massive expansion of this new sub-lineage occurred after the acquisition of E1-I317V in other neighboring and remote regions in 2014–2020. Additionally, the phylogenetic tree indicated that independent clades formed according to the geographical regions and introduction timing. The present results using all available partial or full sequences of the recent CHIKVs emphasized the dynamics of the IOL sub-lineages in the Indian subcontinent, Southeast Asia, and Eastern Africa.

## 1. Introduction

Chikungunya virus (CHIKV) is a member of the genus *Alphavirus*, family *Togaviridae,* and causes fever, arthralgia, and rash in humans. The viral genome is a single positive-stranded RNA of 11–12 kb in length consisting of 5′ and 3′ untranslated regions (UTRs) and two open reading frames (ORFs) of nonstructural proteins nsP1-4 and structural polyproteins capsid, E3, E2, 6K, and E1. The first CHIKV was isolated during an outbreak in Tanzania in 1953. Nowadays, CHIKV is phylogenetically classified into three major genotypes: East/Central/South/African (ECSA), West African (WA), and Asian, named after the location where these genotypes were first recognized [1]. Asian CHIKV that had diverged from ECSA once emerged in South Asia in the 1960s and subsequently spread to Asia [1]. In 2005, CHIKV re-emerged in these regions as the ECSA genotype that was later referred to as the Indian Ocean Lineage (IOL). The IOL CHIKV caused epidemics and consequent outbreaks during the last decade. On the other hand, in 2013, an Asian CHIKV emerged in the Americas, starting from St. Martin in the Caribbean, and spread through the South and Central Americas. Previously, CHIKV was transmitted mainly through *Aedes aegypti* [2]. However, an alanine-to-valine mutation at position 226 within the E1 glycoprotein (E1-A226V) in IOL CHIKV was shown to increase viral replication in *Aedes albopictus* [3]. For this reason, IOL CHIKV might widely spread to where this mosquito is abundant, particularly Thailand, Singapore, and Malaysia in Asia, and even in temperate climate countries such as Italy and France in Europe.

However, IOL CHIKV of the most recent outbreaks during 2016–2017 in India, Pakistan, and Bangladesh lacked E1-A226V. Instead, they carried two novel mutations, a lysine-to-glutamic acid mutation at position 211 within the E1 glycoprotein (E1-K211E) and a valine-to-alanine mutation at position 264 within the E2 glycoprotein (E2-V264A) [4,5,6]. We previously reported that the IOL with these mutations clustered to a novel sub-lineage [6]. IOL sub-lineage E1-K211E/E2-V264A shortly spread from Bangladesh to Thailand in 2017, resulting in outbreaks with tens of thousands of CHIKV-suspected cases. Besides the above-mentioned countries, IOLs carrying E1-K211E and E2-V264A was reported in Italy in 2016–2017 [7], Myanmar, and China in 2019 [8], and even in Eastern Africa, including Kenya, Sudan, and Djibouti in 2014–2019 [9,10,11,12].

To understand the diversity and evolution of the current IOL sub-lineage E1-K211E/E2-V264A, IOL sequences with these mutations were collected in the Virus Pathogen Resource (ViPR; https://www.viprbrc.org) (accessed on 1 August 2021) [13]. In addition, nearly whole genome sequences of Maldives CHIKV collected during the outbreak in 2019 [14] and those of Bangkok CHIKV in 2020 were determined, and then phylogenetic and molecular clock analyses were performed. In this paper, it is shown that IOL sub-lineage E1-K211E/E2-V264A emerged around 2008, likely in India, and caused sporadic outbreaks in India in 2010 and Kenya in 2016. After this, the IOL sub-lineage acquired an additional mutation of an isoleucine-to-valine mutation within E1 (E1-I317V), and it spread widely to several regions, including the Indian subcontinent, Eastern Africa, and Southeast Asia during 2014–2020.

## 2. Materials and Methods

### 2.1. CHIKV Samples

Sixteen existing, previously published CHIKV real-time RT-PCR-positive sera were obtained from Indira Gandhi Memorial Hospital, the Maldives in 2019 [14,15] and 10 newly collected, also CHIKV real-time RT-PCR-positive sera in the Bangkok Hospital for Tropical Diseases in 2020 were used. The study was conducted according to the Declaration of Helsinki, and ethical approval was obtained from the National Health Research Committee in the Maldives. The Ethics Committee of the Faculty of Tropical Medicine, Mahidol University, approved the protocol (Certificate of Ethical Approval No. MUTM 2020-009-01 and 2020-010-01).

### 2.2. CHIKV Genome Sequencing

Twenty-five μL of CHIKV positive sera with low viral load (ct above 16) were used to isolate virus strains in C6/36 cells in order to increase the number of the virus using the previously described protocol [6]. For genome sequencing, total RNA was extracted either from cell culture supernatant or directly from the original sera with high viral titers (ct less than 15) using the QIAamp viral RNA mini kit (Qiagen, Hilden, Germany) and then subjected to PCR amplification [6]. The library was prepared using an Illumina Nextera XT kit (Illumina, San Diego, CA, USA), and the paired-end of the 2 × 250 bp sequencing reaction was conducted using the Miseq platform (Illumina, San Diego, CA, USA). The forward and reverse short reads were aligned to the reference strain (MF773566 Bangladesh 2017) using the map to reference command in CLC Genomics Workbench software version 20 (Qiagen, Aarhus, Denmark). The consensus sequences were extracted and deposited in the DNA Data Bank of Japan (DDBJ, http://www.ddbj.nig.ac.jp) (accessed on 13 December 2021) with the access number LC664141-LC664166 (Table 1). Newly generated CHIKV sequences were trimmed at 5′UTR and 3′UTR to obtain the ORFs region with the untranslated junction region (complete coding sequence; CDS; 11,237 bp). The identities of nucleotides and amino acids of the CHIKV ORFs and the corresponding nonstructural and structural protein sequences, respectively, were determined using compute distance in Mega version 11 [16]. The sequence similarity to CHIKV in the database was examined in the NCBI Blastn suite [17]. To determine genotypes of our newly obtained CHIKV, the maximum likelihood (ML) tree of our sequences, the reference sequences of WA, Asian, ECSA, IOL, and the related sequences from Blast results, were constructed in IQ-TREE [18] (Supplementary Figure S1).

### 2.3. Data Collection and Phylogenetics

The CHIKV sequences were collected using the ViPR database by filtering the geography and time option as the retrieval date of August 2021 [13]. Genbank was also used to trace the publication of the sequences and the PopSet data of the studies. To analyze the presence or absence of the mutations at E1-211, E1-226, and E2-264, 765 IOL CHIKV nucleotide sequences (Supplementary Table S1) covering at least the E2 to E1 region were aligned and translated to amino acids using AliView v1.26 [19]. In order to mitigate possible temporal inference localization errors in the spatiotemporal analyses using a Bayesian phylogenetic methodology, the sequences retrieved from the ViPR database were curated by the following criteria: (1) IOL sequence with E1-K211E and E2-V264A representing multiple geographical regions collected from 2010 to present or the earliest IOL strains in 2004–2005 [1] and their descendant strains collected from 2004 to 2010; (2) sequences covering at least 10,000 bp of CDS region; (3) sequences after removal of the laboratory strains, those with ambiguous nucleotides, or duplicated ones detected by the ML tree analysis and TempEst v1.5.3; (4) sequences after removal of the recombinant screened using GARD [20]. After the curation, the dataset of 271 IOL CHIKV CDS sequences (26 of these were generated in the present study, and the others were collected from the public database, Supplementary Table S3) was finally prepared, and the initial ML tree was constructed using IQ-TREE [18]. The evolutionary temporal signal of the dataset was further inspected in TempEst v1.5.3 prior to the molecular clock analysis (Supplementary Figure S2). The time-scaled tree for the IOL CHIKV sequences was reconstructed using a Bayesian Markov chain Monte Carlo (MCMC) method provided in the BEAST package v1.10.4 under GTR + F + I + G4 with an uncorrelated lognormal clock and Bayesian Skygrid, as described in the previous study [6]. The grid point was set at 16. Four independent runs of MCMC were carried out for 50,000,000 generations each, with sampling every 5000 generations, and they were checked for convergence and the effective sample sizes in Tracer v1.7.1. All runs were combined in LogCombiner v1.10.4. Time of the most recent common ancestor (tMRCA) and its 95% highest probability density 215 (95% HPD) were expressed as a year and parts per 100 of the year, which was subsequently converted to a respective month. The maximum clade credibility (MCC) tree was generated using TreeAnnotator v1.10.4 and visualized in FigTree v1.4.4. The nonsynonymous mutations specific to lineage or clade were investigated in the alignment of amino acid translation prepared in AliView v1.26. To investigate the evolution in the E1 region, another ML tree of 962 sequences of E1 retrieved from the ViPR database was constructed using IQ-TREE (Supplementary Figure S3).

### 2.4. Selection Analysis

The dataset of 271 CDS sequences of IOL CHIKV was trimmed to two datasets of the nonstructural polyprotein ORF (7473 bp) and structural polyprotein ORF (3744 bp). These datasets were used for selection pressure analysis implemented in HyPhy using four methods: a mixed-effects model of evolution (MEME), fast, unconstrained Bayesian approximation (FUBAR), single-likelihood ancestor counting (SLAC), and fixed-effect likelihood (FEL) for the site-specific selection.

## 3. Results

### 3.1. CHIKV Obtained in the Present Study

A total of 26 CHIKV sequences of nearly the whole genome, including 16 Maldives strains from the 2019 outbreak and 10 Thailand strains from the 2020 outbreak, were generated (Table 1). CHIKV genomes shared a high degree of similarity since the nucleotide and amino acid identities among Maldives strains were 99.93–99.99% and 99.84–100%, respectively, while those among Thailand strains were 99.68–99.97% and 99.72–100%, respectively (Supplementary Table S2). To identify the most similar strain to the newly obtained virus in the database, each sequence was used in the NCBI Blastn suite. All Maldives strains showed the highest similarity to an Indian CHIKV collected in June 2019 in Kerala, the southern state (MW042255.1), with 99.85–99.88% nucleotide identities. On the other hand, the Bangkok strains BHTD20-WM05, 14, and 19 were most closely related to the 2019 strains from Bangkok and its neighboring city Samut Sakhon (LC802269 and MT495608, respectively) with 99.85–99.95% nucleotide identities; BHTD20-WM34 and 37 were most closely related to a Southern Thailand strain 2018 (MK468801) with 99.88–99.93% nucleotide identities. Interestingly, the remaining Bangkok strains BHTD20-WM27, 29, 40, 44, and 48 showed the highest nucleotide identities (99.89–99.91%) to the Chinese CHIKV returned from Myanmar in 2019 (MT668625). All newly obtained CHIKV sequences were genotyped as ECSA and belonged to IOL with the related strains from neighboring areas (Supplementary Figure S1). In addition, the obtained CHIKV sequences from the Maldives and Thailand carried the mutations E1-K211E and E2-V264A (Supplementary Table S1).

### 3.2. Distribution of Indian Ocean Lineage Carrying E1-K211E and E2-V264A of CHIKV ECSA

To determine the distribution of novel mutations of E1-K211E and E2-V264A, CHIKV nucleotide sequences available in the ViPR database retrieved on 1 August 2021 were investigated. Seven hundred and sixty-five IOL CHIKV sequences covering at least the E2 to E1 region were analyzed. Among them, 427 showed the presence of E1-K211E and E2-V264A (Supplementary Table S1). In Table 2, the earliest detection of E1-K211E and E2-V264A mutation was observed in November 2009–January 2010 in Singapore and India. Initially, the detection of this variant was limited within these two countries during the period of 2009–2013. Although the Singapore strain MH647212 was the earliest strain with E1-K211E and E2-V264A, the majority of Singapore strains in 2010–2012 were sporadic and imported cases [21]. On the other hand, the Indian strains were reportedly associated with local outbreaks, particularly those in the northern part, with the highest number of sequences recorded in 2010 [22]. Later, IOL sub-lineage E1-K211E/E2-V264A was first detected in the new region, particularly Kenya, in 2014–2015 and continually observed until 2018. In 2016, the number of reported IOL sub-lineage E1-K211E/E2-V264A sequences increased substantially in India and was subsequently found in neighboring countries such as Pakistan in 2016–2017 and Bangladesh in 2017, where massive outbreaks were reported in those years. The distribution of the IOL carrying E1-K211E and E2-V264A became remarkable in 2018–2019 when this IOL was widely reported in multiple regions and countries, including the south of the Indian subcontinent (Maldives), Africa (Sudan, Djibouti), Southeast Asia (Myanmar, Thailand, and Malaysia), and East Asia (China and Taiwan). Consequently, local transmission occurred in many of these regions. Additionally, the coincidence of this IOL new sub-lineage in non-endemic countries was frequently reported during the peak of sequence detection in epidemic countries (Table 2).

### 3.3. Evolutionary Dynamics of the Indian Ocean Lineage of CHIKV ECSA

**Figure 1:**
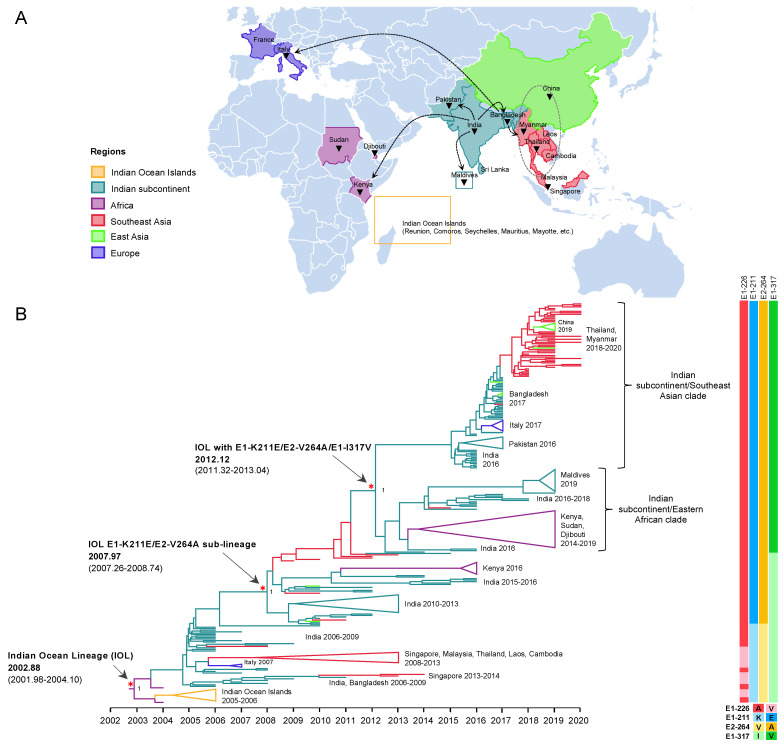
Evolution and geographic distribution of IOL. (**A**) A map showing the location of IOL CHIKV whole-genome sequences analyzed in the present study. The black triangles indicate countries in which IOL sub-lineage E1-K211E/E2-V264A was circulating. Dotted arrows indicate probable transmission routes. (**B**) The maximum clade credibility tree (MCC) for 271 Indian Ocean Lineage (IOL) of CHIKV open reading frame sequences constructed by BEAST under uncorrelated lognormal clock and GTR + F + I + G4. The emergence of the IOL and IOL sub-lineage E1-K211E/E2-V264A with the time of the most recent common ancestor (tMRCA) and its 95% highest probability density (95% HPD) are indicated by red asterisks and arrows. The numbers of posterior probability (PP) support are shown adjacent to the key nodes. The Indian subcontinent/Eastern African clade and Indian subcontinent/Southeast Asian clade are indicated in the right bracket. Triangular clades represent collapsed sequences indicated to the right. The branch color corresponds to the geographic region indicated. The timescale in years is shown on the x-axis at the bottom. The amino acid mutations specific to each lineage are shown on the right, and the color corresponds to the amino acid indicated below.

To investigate the evolution of IOL ECSA of CHIKV in 2004–2020, a dataset of nearly full length 271 CDS was constructed, which consisted of sequences from the earliest IOL strains detected in coastal Kenya and Comoros in 2004 [23] to the recent outbreak in 2020, covering all the geographic regions of IOL CHIKV epidemics including Africa, the Indian subcontinent, Southeast Asia, East Asia (Myanmar and Thailand border of China), and Europe (Italy and France). The dataset exhibited a strong positive correlation between genetic divergence and sampling time (R^2^ = 0.91) (Supplementary Figure S2). The time-scaled MCC tree inferred in BEAST showed the evolutionary dynamics of IOL sub-lineages of CHIKV ECSA across the countries surrounding the Indian ocean over time (Figure 1). The tree topology showed two major spreads of the IOL. The tMRCA for the first spread was November 2002 (2002.88) with an interval of December 2001–February 2004 (95% HPD of 2001.98–2004.10). The long tMRCA interval reflected the small number of genome sequences collected at this period. The earliest IOL originated from coastal Africa (Kenya HQ456254 and HQ456255) and was separately introduced to the Indian Ocean and Indian subcontinent. IOL strains that circulated in the Indian subcontinent descended to several clades, such as Southeast Asian and Italian clades. On the other hand, the second major spread of the IOL was associated with E1-K211E/E2-V264A, forming a monophyletic clade distinct from the previously spread IOL. This new IOL sub-lineage emerged in December 2007 (2007.97) with an interval of April 2007–September 2008 (95% HPD: 2007.26–2008.74) by the basal sequence of the sub-lineage observed in India and was re-introduced not only to the same regions, as in the previous spread in Southeast Asia, but also to Eastern Africa. Furthermore, IOL sub-lineage E1-K211E/E2-V264A later diverged into two distinct clades in February 2012 (2012.12) with an interval of April 2011–January 2013 (95% HPD: 2011.32–2013.04), with a posterior probability (pp) support at 1.

#### 3.3.1. The Emergence of IOL Sub-Lineage E1-K211E/E2-V264A

**Figure 2:**
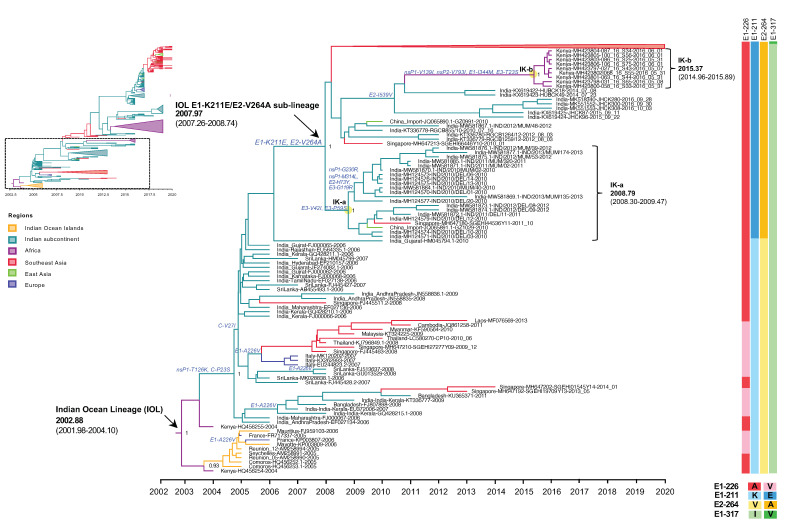
An enlarged view of the IOL MCC tree showing the root IOL and the early IOL sub-lineage E1-K211E/E2-V264A corresponding to the dotted square region of the top-left inset of reduced Figure 1B. Root and sub-lineage E1-K211E/E2-V264A of IOL with the most recent common ancestor (tMRCA) and 95% highest probability density (95% HPD) are indicated by arrows. The numbers of posterior probability (PP) support and amino acid substitutions are shown adjacent to the ancestral key nodes. The IOL variants are indicated in brackets. The yellow circle indicates the key node of IOL clades. The IOL clade, tMRCA, and 95% HPD are indicated in brackets. The branch color corresponds to the geographic region indicated. The timescale in years is shown on the x-axis at the bottom. The amino acid mutations specific to each lineage are shown on the right, and the color corresponds to the amino acid indicated below.

Our molecular clock analysis suggested that the IOL emerged in the Indian subcontinent in September 2004 (2004.73) with an interval of June 2004–February 2005 (95% HPD: 2004.48–2005.10), then spread and circulated in India, Sri Lanka, and Bangladesh, subsequently descended to the new clades, and was distributed to wider geographic regions. The tMRCA for the Southeast Asian clade, including sequences from Singapore, Malaysia, Thailand, Laos, and Cambodia, was estimated to be July 2007 (2007.50) with an interval of February–November 2007 (95% HPD: 2007.16–2007.88), whereas that for the Italian clade was July 2006 (2006.52) with an interval of February 2006–January 2007 (95% HPD: 2006.14–2007.0). During the first emergence of the IOL, the remarkable adaptive mutation E1-A226V was identified as it enhanced IOL CHIKVs replication in *Aedes albopictus*. Co-circulation of the IOL E1-A226V variant with E1-226A virus was primarily observed in the Indian Ocean clade and the South Asian clade during 2005–2013 (Figure 2). The circulation of the IOL E1-A226V variant was predominant in the Southeast Asian clade and the Italian clade, whereas in India, a sporadic outbreak of the IOL E1-A226V variant and E1-226A virus continued through 2006–2014. The investigation of CHIKV sequences in the database showed that the sequence presenting E1-A226V was totally absent from India in 2014 (Supplementary Table S1).

Since 2010, the proportion of E1-K211E/E2-V264A in the background of E1-226A has increased year by year (Table 2). As described above, E1-K211E/E2-V264A IOL was first detected in November 2009 in Singapore, but the majority of strains reported in Singapore in 2009–2012 were either sporadic or imported strains [21]. In the same period, E1-K211E/E2-V264A IOL was also detected in India in January 2010 [22]. Among E1 sequences, the mutation E1-K211E was initially observed in Puducherry, the southern state of India, in 2006 [24]. Since there was an insufficient number of full sequences but a sufficient number of E1 sequences from India before 2010, another phylogenetic analysis was conducted using 962 E1 sequences to investigate the evolution of E1-K211E (Supplementary Figure S3). The tree topology showed that not only nearly full-length CHIKV genomic sequences carrying E1-K211E/E2-V264A (Figure 1) but also CHIKV E1 sequences carrying E1-K211E in the background of E1-226A (Supplementary Figure S3) formed a distinct clade as the new sub-lineage. As described above, the IOL sub-lineage E1-K211E/E2-V264A emerged in December 2007 (2007.97) with an interval of April 2007–September 2008 (95% HPD: 2007.26–2008.74). In the early period, the E1-K211E/E2-V264A IOL evolved into two clades: the India-Kenya clades a (IK-a) and b (IK-b). IK-a consisted of sequences from India in 2010–2013 that shared a tMRCA in October 2008 (2008.79) with an interval of April 2008–June 2009 (95% HPD: 2008.30–2009.47, whereas IK-b was a cluster of Kenyan strains in 2016 shared tMRCA in May 2015 (2015.37) with an interval of December 2014–November 2015 (95% HPD: 2014.96–2015.89). Although IK-b Kenya strains were most closely related to the Indian strains 2014 (KX619422 and KX619423), they shared E1-K211E and E2-V264A in common, the long branch between IK-b and New Delhi 2014 suggested possible missing sampling of circulating strains in this gap. In addition, there were nonsynonymous mutations (nsP1-V139I, nsP2-V793I, E1-I344M, E3-T23S) specific to the IK-b clade, indicating a unique evolution of the Kenya 2016 viruses [25].

#### 3.3.2. The Expansion of IOL Sub-Lineage E1-K211E/E2-V264A

**Figure 3:**
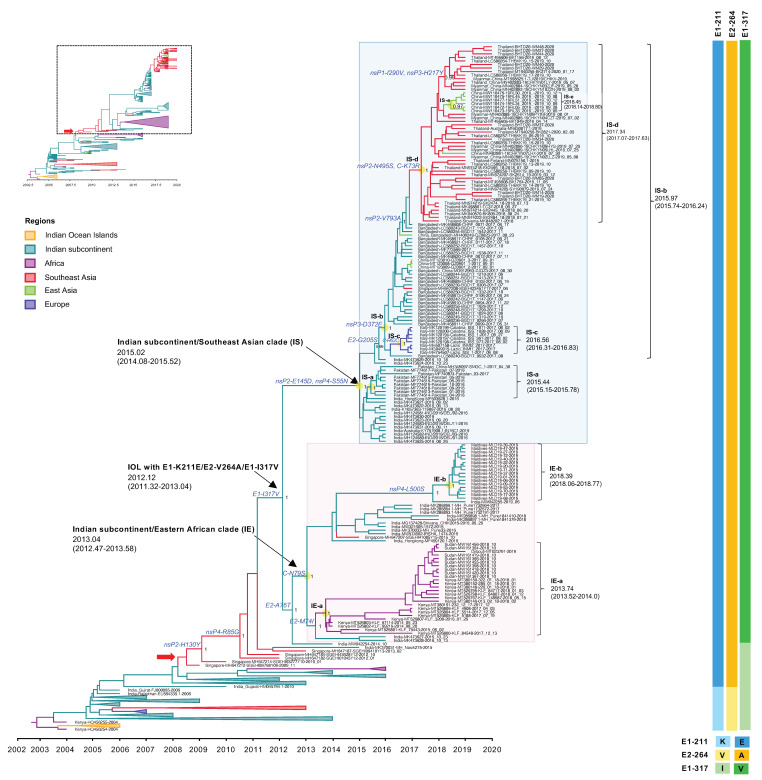
An enlarged view of the IOL MCC tree showing IOL sub-lineage E1-K211E/E2-V264A and E1-I317V of the IOL MCC tree corresponding to the dotted square region of the top-left inset of reduced Figure 1B. The Indian subcontinent/Eastern African (IE) clade and Indian subcontinent/Southeast Asian (IS) clade with the most recent common ancestor (tMRCA) and 95% highest probability density (95% HPD) are indicated by arrows and the pink- and light blue-shaded boxes, respectively. The number of posterior probability (PP) support and amino acid substitutions are shown adjacent to the ancestral key nodes. IOL clades, tMRCA, and 95% HPD are shown in brackets. The branch color corresponds to the geographic region indicated. The timescale in years is shown on the x-axis at the bottom. The amino acid mutations specific to each lineage are shown on the right, and the color corresponds to the amino acid indicated below.

The expansion of the IOL sub-lineage E1-K211E/E2-V264A was observed in recent outbreaks after 2015. This new IOL sub-lineage India and Pakistan strains circulating in 2016 spread rapidly to Bangladesh in 2017 and from Bangladesh to Myanmar, Thailand, and China in 2018–2020. Moreover, this IOL sub-lineage also spread from India to the Maldives and Kenya, Sudan, and Djibouti in 2014–2019. In Figure 3, the phylogenetic analyses show that those sequences were clustered to a monophyletic clade at a posterior probability support of 1 and shared the tMRCA at 2012.12, with a substitution rate of 1.22 × 10^−3^ substitutions/site/year (s/s/y). Besides the mutations E1-K211E/E2-V264A, this clade shared an additional amino acid substitution of E1-I317V specific to the recent IOL 2014-2020. The phylogenetic tree in Figure 3 show that IOL sub-lineage E1-K211E/E2-V264A with E1-I317V independently evolved into two major phylogenetic clades corresponding to different geographical regions, including the Indian subcontinent/Eastern African clade (IE clade) and the Indian subcontinent/Southeast Asia clade (IS clade), which were arbitrarily named according to the virus-circulating regions.

The Indian subcontinent/Eastern Africa clade (IE clade) contained viruses from India 2016–2019, Kenya 2014–2018, Sudan 2018, Djibouti 2019, and Maldives 2019. The tMRCA of this clade was estimated at January 2013 (2013.04) with an interval of June 2012–July 2013 (95% HPD: 2012.47–2013.58), with a substitution rate of 1.88 × 10^−3^ (2.86 × 10^−3^–4.51 × 10^−3^) s/s/y. The IE viruses had C-N79S and E2-A76T as the common amino acid substitutions among the clade. In addition, there were two distinct subclades within this clade with different geographic regions of their sampling site: (1) Eastern Africa subclade or (IE-a) and (2) Maldives subclade (IE-b) that originated in September 2013 (2013.74) with an interval of July 2013–January 2014 (95% HPD: 2013.52–2014.0) and May 2018 (2018.39) with an interval of January–October 2018 (95% HPD: 2018.06–2018.77), respectively. The IE-a subclade contained Kenyan isolates 2014–2018 together with viruses from neighboring countries, including Sudan in 2018 and Djibouti in 2019. IE-a viruses shared the mutation of E2-M74I. On the other hand, the IE-b subclade was limited to viruses only from the Maldives collected in 2019. The most related strain to IE-b was India strain 2019 (MW042255), suggesting that the new sub-lineage had spread from India to the Maldives, circulating widely to the south of the Indian subcontinent. Those sequences shared the unique amino acid mutation of nsP4-L500S.

The Indian subcontinent/Southeast Asia clade (IS clade) was a large monophyletic clade comprising isolates from India, Pakistan, and Bangladesh in 2016–2017, and Thailand, Myanmar, and China (adjacent cities to Myanmar) in 2018–2020. The tMRCA was estimated at January 2015 (2015.02) with an interval of January 2014–July 2015 (95% HPD: 2014.08–2015.52) with a substitution rate of 6.04 × 10^−4^ (3.11 × 10^−4^–9.51 × 10^−4^) s/s/y. The analysis suggested an expansion route of IOL sub-lineage -E1-K211E/E2-V264A with E1-I317V virus that reached the adjacent regions including Pakistan and Bangladesh in the 2016–2017 epidemic from India, especially northern India, particularly Delhi and Maharashtra, located in the south of Delhi. The phylogenetic tree indicated that the IS-a subclade arose in Pakistan around June 2015 (2015.44) with an interval of February–October 2015 (95% HPD: 2015.15–2015.78) and that the IS-b subclade emerged in Bangladesh in December 2015 (2015.97) with an interval of September 2015–March 2016 (95% HPD: 2015.74–2016.24). The IS-c subclade was composed of Italian viruses that arose in July 2016 (2016.56) with an interval of April–October 2016 (95% HPD: 2016.31–2016.83). Additionally, the IS-b subclade consisted of the descending strains circulating during 2018–2020 in Southeast Asia, including Thailand, Myanmar, and China (cities adjacent to Myanmar), forming a clade IS-d separate from the Bangladesh strains. The re-introduction of the IOL to these regions occurred around May 2017 (2017.34) with an interval of January–August 2017 (95% HPD: 2017.07–2017.63). Moreover, local transmission in China was observed in the IS-e subclade. In addition, the IS clade was defined by amino acid mutations of nsP2-E145D and nsP4-S55N, whereas the IS-d subclade was defined by nsP2-N495S and C-K73R.

### 3.4. Selection Analyses

The dataset of 271 IOL CHIKV sequences was screened for positive selection using different individual site models. Seven codon sites were identified under positive selection by at least two methods (Table 3). Of these, two sites were within nonstructural polyproteins, codon 171 in nsP1 and codon 665 in nsP2, which corresponded to R171Q and H130Y. Notably, nsP2-H130Y was observed in viruses of IE and IS clades collected during 2014–2020. Furthermore, five codons were within structural polyproteins in C, E2, 6K, and E1 (Table 3). In particular, codon 471 with Q146R substitution at E2 showed significant values by all four methods.

## 4. Discussion

In the present study, IOL CHIKV sequences with time spanning from 2004 to the present were analyzed to investigate the evolution of the IOL for a better understanding of the current IOL genetic diversity. The first emergence of the IOL of CHIKV genotype ECSA was in late 2002, consistent with the previous studies [1]. Based on the available deposited sequences in the database, the earliest IOL that originated in coastal Kenya potentially caused an impact on the spread and transmission over several countries in the Indian Ocean, Indian subcontinent, and Southeast Asia. Notably, an adapted CHIKV variant carrying A226V in E1 that enhances viral replication in *Aedes albopictus* was identified [3]. The IOL-E1-226V variants dominated successfully over other variants, especially where *Aedes albopictus* was abundant, such as in Kerala in India in the 2007 outbreak [26]. Consequently, the IOL-E1-226V CHIKV was introduced to Singapore, Thailand, and Malaysia in 2008, resulting in the subsequent outbreaks through 2008–2013. The present investigation of IOL sequences carrying E1-226V showed that E1-226V was totally absent from India after 2014 (Supplementary Table S2). Although both IOL E1-226A and E1-226V variants were detected during the continuous outbreaks in India in 2007–2010, IOL E1-226A was the major strain at that time [27,28,29,30].

After the initial major outbreaks, Indian IOL has retained E1-226A but gained additional mutations of E1-K211E and E2-V264A. As described above, the earlier presence of E1-K211E/E2-V264A IOL was reported in early 2010, particularly in Tamil Nadu, Andra Pradesh, and Delhi in India [22,29,31]. The present phylogenetic analysis showed that E1-K211E/E2-V264A IOL formed a distinct monophyletic clade with high posterior probability support around 2008, establishing the new sub-lineage of IOL consisting of the descended clades of IOL 2010–2020 strains (Figure 1 and Figure 2). Notably, the E1 and E2 glycoproteins mediated cell fusion and entry [32]. They formed an E1-E2 heterodimer on the viral surface [33]. The combination of E1-K211E and E2-V264A mutations was proposed as the viral adaptation to *Aedes aegypti* by increasing infectivity, dissemination, and transmission in this mosquito species [5]. *Aedes aegypti* was present in several areas over India instead of *Aedes albopictus* [34,35]. Evidently, the IOL transmission in India had expanded widely, from 13 states in the first emergence in 2005–2006 to 30 states/union territories in 2019 [35].

In terms of the amino acid variation at 211 in E1 among CHIKV genotypes, an E1-K211E was observed in the IOL variant in 2006 in Kerala and Puducherry, India [29]. On the other hand, E1-211E was highly conserved among Asian genotypes in both the Asia lineage that circulated since 1963 and the Caribbean lineage that emerged recently in 2013. E1-211E conferred resistance against neutralization activity in human sera [36]. Unfortunately, the exact beginning of the E2-V264A mutation is unclear since prior to 2010, only the E1 region was commonly sequenced. However, the earliest E2-V264A appeared possibly in January 2010 in Tamil Nadu as a secondary mutation after the virus acquired E1-K211E since the combination of the wild type E2-264V and E1-K211E was detected in December 2009 in Hyderabad, Andhra Pradesh [29]. Vector competence and adaptation were driven where *Aedes aegypti* and *Aedes albopictus* coexisted. The mutation E1-K211E with E2-V264A and the E1-A226V with E2-L210Q or E2-K252Q enhanced virus fitness in *Aedes aegypti* and *Aedes albopictus*, respectively [5,37,38]. Accordingly, India is supposed to be the origin of the IOL sub-lineage E1-K211E/E2-V264A. However, it should be noted here that the previous CHIKV outbreak in India led the national surveillance program, which might have resulted in more perceptible database deposition of the viral sequences than in other countries, potentially causing unrecognized influences in our tracing.

At first, the earliest E1-K211E/E2-V264A IOL strain sparsely circulated in Delhi, Mumbai, and Kerala in India since 2010 [25]. Then, the wide expansion of IOL sub-lineage E1-K211E/E2-V264A occurred after the virus acquired the mutation of E1-I317V associated with the later outbreaks. The IOL with these three mutations clustered into the distinct clade that shared a tMRCA in 2012, while the earliest detection of this clade virus was around October 2014 in Kerala, the southern state of India [39]. However, the individual branches, including Singaporean strains [21] and Indian strains [40] observed between E1-K211E/E2-V264A and E1-K211E/E2-V264A/E1-I317V clusters indicated inadequate sequence sampling, especially during the inter-epidemic (or inter-outbreak) period during which IOL variants were possibly diverse.

Shortly after its emergence in 2012, E1-K211E/E2-V264A/E1-I317V IOL separated into geographically associated clades. In 2013, this variant was introduced to coastal Kenya in Kilifi and Mombasa in 2014 [41]. Interestingly, the re-emergence of CHIKV in Kenya during 2014–2018 was clearly independent of the Kenya 2016 cluster of IK-b clade in Mandera, the northern city of Kenya where *Aedes aegypti* was predominant. Notably, the earliest Kenya strain collected in 2014 was related to Indian 2016 isolates (MK473627-MK473628) and India isolate 2014 (MW042254). This IOL variant was also detected in the ongoing outbreaks in nearby countries Sudan and Djibouti that occurred coincidently with the rise of the suspected CHIKV infections in other Eastern African countries such as Ethiopia [9,10,11,42]. Additionally, the genomes of Maldives CHIKV obtained in the present study were related to Southern India (Kerala and Pune state) and Eastern African strains rather than Northern Indian subcontinent strains defined by the lineage-specific mutations observed in the IE clade. On the other hand, the circulation of E1-K211E/E2-V264A/E1-I317V IOL in the northern parts of the Indian subcontinent was related to CHIKVs in Southeast Asia and even East Asia, particularly China. The early circulation was detected in Delhi, India, and Pakistan during 2016. The phylogenetics suggested that this IOL was disseminated to adjacent countries from Bangladesh to Myanmar, Thailand, and China. Evidently, these IOL variants were related to the outbreak in Myanmar in 2019, which subsequently contributed to an outbreak in Yunnan, China [8,43].

Regarding travel-associated CHIKV transmission to remote regions in the non-endemic area such as East Asia, imported cases were reported in Hong Kong returning from India in 2016 [44], in Shenzhen, China from Pakistan in 2017 [45], in Zhejiang, China from Bangladesh, followed by a small cluster [46] in Europe from Thailand [47]. Interestingly, the introduction of CHIKV to the places with Aedes mosquitos resulted in an autochthonous case or even a small outbreak [7]. However, it was probably limited by insufficient vector availability during the dry season. When ECSA CHIKV invaded Brazil, the virus was imported from Angola by the index case with a travel history that triggered its sustaining circulation [48].

The functional role of the lineage-specific mutation E1-I317V is still less known. The mutation was certainly detected in circulating IOL from 2014 to the present, but there was no evidence of positive selection pressure on this mutation. The study of in vivo pathogenesis among CHIKV lineages showed that there was no difference in the swelling of mouse footpads between the Indian IOL 2010 strain with E1-317I and the Indian IOL 2016 strain with E1-317V [22]. Likewise, a vector competence study comparing Italy 2007 and Italy 2017 strains in *Aedes albopictus*, the localized vector in Italy, showed that both had similar infection and transmission rates [49]. In addition, in the large-scale outbreak of IOL sub-lineage E1-K211E/E2-V264A carrying E1-I317V in Thailand during 2018–2020, *Aedes aegypti* was identified as the primary vector, and viral RNA was also found in another mosquito species, *Culex quinquefasciatus* [50,51]. In Mombasa, Kenya, these two mosquito species might have played a role in the outbreak of IOL sub-lineage E1-K211E/E2-V264A/E1-I317V [41,52]. Whether this mutation has a functional role in viral adaptation to *Aedes aegypti* or other mosquito species needs to be clarified by further studies in the future.

The present study characterized the diversity of IOL lineages, especially the current circulating strains. The limitations of this study are the availability of data and the difficulty inferring the origin of sequences from the database. Since collection probably placed a priority on outbreaks and symptomatic patients, fewer viral sequences were deposited within the inter-epidemic/non-outbreak period. The different sampling strategies or surveillances in each collection location would also generate biases. To better understand the dynamics of the IOL, a sampling schema over a wide range of geography, time, and transmission vectors diversity is important.

## Figures and Tables

**Table 1:** List of CHIKV sequences in the present study.

Strain	Collection Date	Location	Sample Type	Passage History	Accession No.
MLD19-01	30 March 2019	Maldives	isolate	C6/36	LC664141
MLD19-06	31 March 2019	Maldives	isolate	C6/36	LC664142
MLD19-09	30 March 2019	Maldives	isolate	C6/36	LC664143
MLD19-20	24 April 2019	Maldives	isolate	C6/36	LC664144
MLD19-22	1 April 2019	Maldives	isolate	C6/36	LC664145
MLD19-27	3 April 2019	Maldives	isolate	C6/36	LC664146
MLD19-37	7 April 2019	Maldives	serum	no passage	LC664147
MLD19-39	4 April 2019	Maldives	isolate	C6/36	LC664148
MLD19-40	April 2019	Maldives	isolate	C6/36	LC664149
MLD19-47	10 April 2019	Maldives	isolate	C6/36	LC664150
MLD19-53	14 April 2019	Maldives	isolate	C6/36	LC664151
MLD19-68	19 May 2019	Maldives	isolate	C6/36	LC664152
MLD19-70	May 2019	Maldives	isolate	C6/36	LC664153
MLD19-71	May 2019	Maldives	isolate	C6/36	LC664154
MLD19-72	18 June 2019	Maldives	isolate	C6/36	LC664155
MLD19-77	2 August 2019	Maldives	serum	no passage	LC664156
BHTD20-WM05	29 June 2020	Bangkok, Thailand	isolate	C6/36	LC664157
BHTD20-WM14	21 July 2020	Bangkok, Thailand	isolate	C6/36	LC664158
BHTD20-WM19	4 August 2020	Bangkok, Thailand	isolate	C6/36	LC664159
BHTD20-WM27	19 August 2020	Bangkok, Thailand	serum	no passage	LC664160
BHTD20-WM29	20 August 2020	Bangkok, Thailand	isolate	C6/36	LC664161
BHTD20-WM34	26 August 2020	Bangkok, Thailand	isolate	C6/36	LC664162
BHTD20-WM37	28 August 2020	Bangkok, Thailand	serum	no passage	LC664163
BHTD20-WM40	8 September 2020	Bangkok, Thailand	serum	no passage	LC664164
BHTD20-WM44	13 September 2020	Bangkok, Thailand	serum	no passage	LC664165
BHTD20-WM48	25 September 2020	Bangkok, Thailand	serum	no passage	LC664166

**Table 2:** Number of CHIKV sequences with E1-K211E and E2-V264A by region/country and year of collection.

Year	Local Strain		Travel-Associated Strain	
	Region	Location	Region	Reported Country (Origin Country)
2009	Southeast Asia	Singapore * 1 (1)		
2010	Indian subcontinent	India 20 (15)	Europe	France (India) 1 (0)
	Europe	France 1 (0)	East Asia	China (India) 4 (4)
	Southeast Asia	Singapore * 4 (4)		
2011	Indian subcontinent	India 8 (5)		
	Southeast Asia	Singapore * 2 (2)		
2012	Indian subcontinent	India 16 (10)		
	Southeast Asia	Singapore * 3 (3)		
2013	Indian subcontinent	India 6 (6)		
	Southeast Asia	Singapore * 2 (2)		
2014	Indian subcontinent	India 4 (4)		
	Africa	Kenya 2 (2)		
2015	Indian subcontinent	India 5 (5)		
	Southeast Asia	Singapore * 1 (1)		
	Africa	Kenya 1 (1)		
2016	Indian subcontinent	India 27 (27), Pakistan 8 (7)	Pacific	Australia (India) 1 (1)
	Africa	Kenya 15 (12)	East Asia	Hong Kong (India) 2 (2)
2017	Indian subcontinent	India 4 (4), Pakistan 5 (1), Bangladesh 37 (37)	East Asia	China (Pakistan) 1 (1), (Bangladesh) 2 (2)
	Africa	Kenya 5 (5)	Pacific	Australia (Bangladesh) 1 (1)
	Europe	Italy 10 (10)		
	Southeast Asia	Singapore * 1 (1)		
2018	Indian subcontinent	India 2 (2)	Europe	Slovenia (Thailand) 1 (1)
	Africa	Kenya 19 (19), Sudan 80 (80)		
	Southeast Asia	Thailand 15 (15)		
2019	Indian subcontinent	India 1 (1), Maldives 16 (16)	Pacific	Australia (Thailand) 1 (1)
	Africa	Djibouti 1 (1)	East Asia	China (Myanmar 10 (10), Thailand 1 (1)) Taiwan (Myanmar 7 (0), Thailand 4 (0), Malaysia 1 (0))
	Southeast Asia	Thailand 29 (29)	Europe	Finland (Thailand) 2 (2)
	East Asia	China 7 (7), Taiwan 8 (0)		
2020	Southeast Asia	Thailand 21 (21)		

The numbers in brackets indicate those with the whole genome sequences, * Asterisks indicate suspected travel-associated strains.

**Table 3:** Evidence of positive selection on the IOL CHIKV ORFs inferred using individual site models.

Codon Site	MEME *p* < 0.1	FUBAR pp > 0.9	FEL *p* < 0.1	SLAC *p* < 0.1	Amino Acid Substitution	Sequences with Derived Amino Acid State
**Nonstructural proteins**						
171	**0.00**	**0.999**	**0.002**	**0.006**	nsP1-R171Q	Comoros 2005: HQ456252 Italy 2007: MK120202, KX262993 Sri Lanka 2006: AB455493 India 2016: MW321606, MG137428; 2017: MK286893 Kenya 2016: MH423803; 2017: MT380161
665	0.12	**0.946**	**0.095**	0.131	nsP2-H130Y	Singapore 2009: MH647212; 2010: MH647214 2012: MH647182, MH647184; 2013: MH647187 India 2014: MW042254; 2015: MK370031 IE clade virus *, IS clade virus *
**Structural proteins**						
24	**0.09**	0.893	**0.069**	0.296	C-T24A	Laos 2013: MF076569 Djibouti 2019: MT023791 Thailand 2019: LC580269; 2020: BHTD20-WM14, BHTD20-WM19
471	**0.03**	**0.995**	**0.022**	**0.079**	E2-Q146R	Seychelles 2005: AM258991 Bangladesh 2017: MG912993 Kenya 2018: MT526797 Thailand 2019: MN075150; 2020: BHTD20-WM05
546	0.12	**0.965**	**0.097**	0.237	E2-K221R	India 2012: MW581867 Bangladesh 2017: LC580241, LC580244 Thailand 2019: MN075150; 2020: BHTD20-WM27
795	**0.07**	**0.902**	**0.061**	0.296	6K-A47V 6K-A47G	India 2016: MK473625 Pakistan 2016: MF774613 Singapore 2013: MH647192; 2014: MH647202
813	0.12	**0.946**	**0.093**	0.198	E1-V4A	India 2007: EU372006; 2008: GQ428215; 2009: KT336777 2015: KX619425, KX619424 2016: MK518340, MK551552, MK551553, MK473628 Bangladesh 2008: FJ807898; 2011: KU365371 Singapore 2013: MH647192; 2014: MH647202 Thailand 2020: BHTD20-WM44, BHTD20-WM27, BHTD20-WM48

Positive selection site is highlighted in boldface, *p* = *p*-value, pp = posterior probability, * Virus clades correspond to those in Figure 3.

## Data Availability

The newly obtained sequences were deposited in the DNA Data Bank of Japan (DDBJ, http://www.ddbj.nig.ac.jp) (accessed on 13 December 2021) with the access number LC664141-LC664166 and are available in DDBJ and GenBank. The viral sequences analyzed in the present study were retrieved using ViPR and are shown in Tables S1 and S3.

## Supplementary Materials

The following are available online at https://www.mdpi.com/article/10.3390/microorganisms10020354/s1, Table S1. Amino acid residues at E1-211, E1-226, and E2-264 in 765 IOL CHIKVs analyzed in the present study. Table S2. Percent nucleotide or amino acid identity of CHIKV strains obtained in the present study. Table S3. Nearly full length 271 IOL CHIKV sequence dataset in the present study. Figure S1. Genotype classification of CHIKVs was obtained in the present study. The maximum-likelihood tree of open reading frames (ORFs) were constructed using GTR+F+I with 1000 ultrafast bootstrap replications. The Maldives and Thailand sequences obtained in the present study are labeled with green and blue, respectively. The CHIKV genotypes and a lineage, East/Central/South/African (ECSA), West African (WA), Asian, and Indian Ocean Lineage (IOL) are indicated to the right. Bootstrap support values exceeding 85% are shown adjacent to the branch. Figure S2. Root-to-tip divergence analysis of the dataset of 271 IOL CHIKV CDS sequences. The regression of root-to-tip divergence against date inferred in TempEst v1.5.3 is shown. The R^2^ = 0.91 is shown adjacent to the regression line. The x-intercept (tMRCA) was 2003.82. The slope (rate) was 5.59 × 10^−4^. The date range was 16 years. The color marker represents the regions indicated on the right. Figure S3. The Maximum Likelihood tree for 962 E1 sequences (1317 bp) of Indian Ocean Lineage (IOL) of CHIKV constructed by IQ-TREE under TNe+G4. The combinations of amino acid variations in E1 K211/A226/I317, K211N/A226V/I317, K211/A226V/I317, K211E/A226/I317, and K211E/A226/I317V corresponding to the sequence color black, pink, red, green, and blue, respectively. The number adjacent to the branch indicates the support score of ultrafast bootstrap.
