# Supplementary material for: Spread of a Novel Indian Ocean Lineage Carrying E1-K211E/E2-V264A of Chikungunya Virus East/Central/South African Genotype across the Indian Subcontinent, Southeast Asia, and Eastern Africa
Source: Microorganisms. 2022 Feb 3;10(2):354. doi: 10.3390/microorganisms10020354 (PMC8878743; doi:10.3390/microorganisms10020354)
Supplement: Supplementary file 1 [file microorganisms-10-00354-s001.zip › Micro1535353-Sup-FigureS1-S3.pdf]

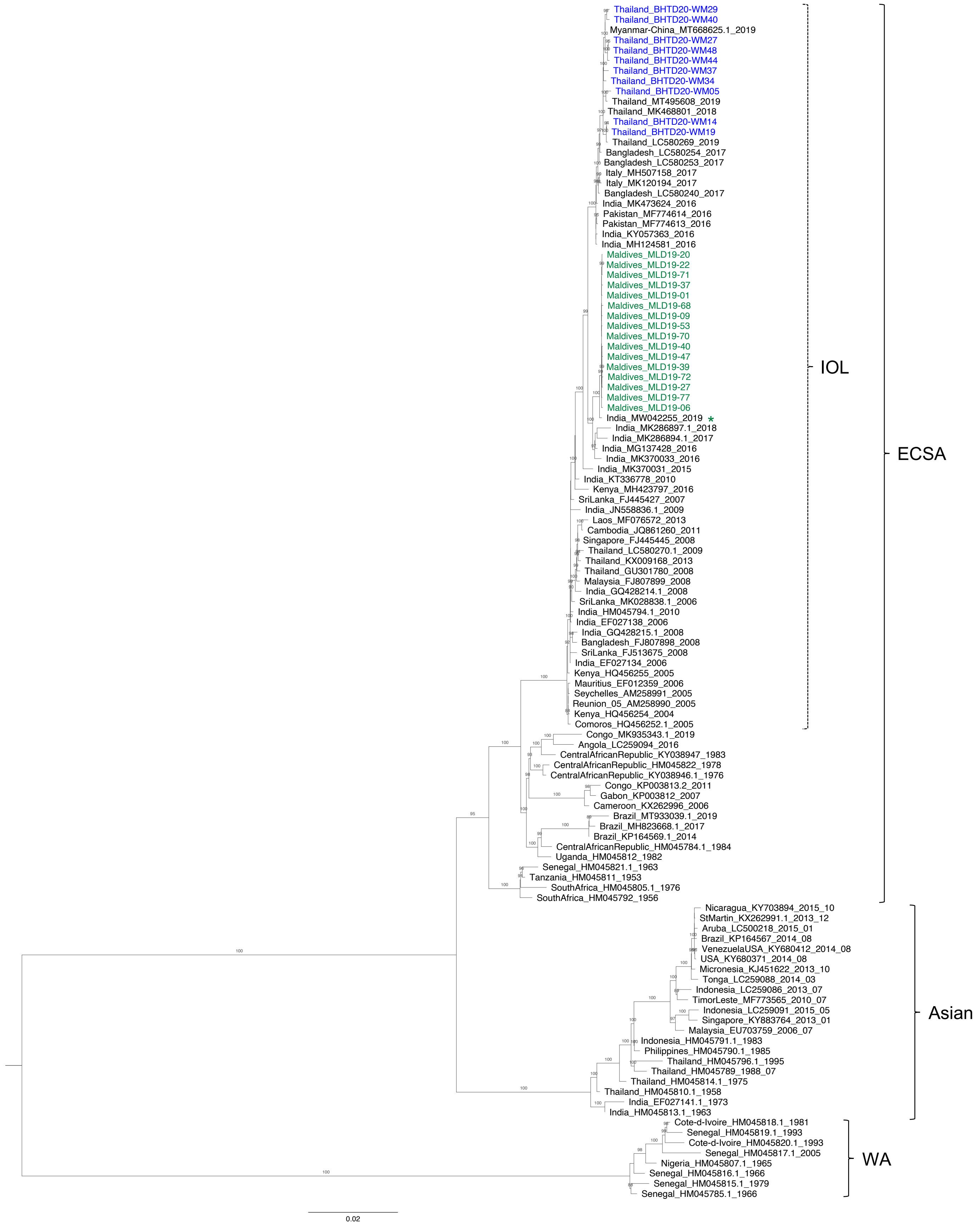

Figure S1. Genotype classification of CHIKVs obtained in the present study. The maximum-likelihood tree of open reading frames (ORFs) were constructed using GTR+F+I with 1000 ultrafast bootstrap replications. The Maldives and Thailand sequences obtained in the present study are labeled with green and blue, respectively. The CHIKV genotypes and a lineage, East/Central/South/African (ECSA), West African (WA), Asian, and Indian Ocean Lineage (IOL), are indicated to the right. Bootstrap support values exceeding 85% are shown adjacent to the branch.

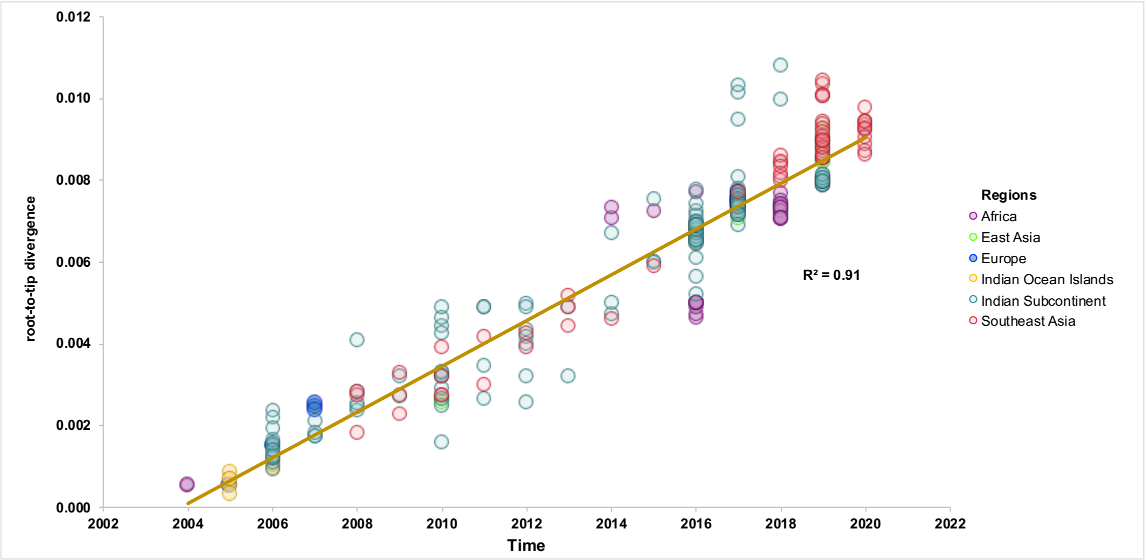

Figure S2. Root-to-tip divergence analysis of the dataset of 271 IOL CHIKV CDS sequences. The regression of root-to-tip divergence against date inferred in TempEst v1.5.3 is shown. The $R^2 = 0.91$ is shown adjacent to the regression line. The x-intercept (tMRCA) was 2003.82. The slope (rate) was 5.59×10^{-4}. The date range was 16 years. The color marker represents the regions indicated on the right.

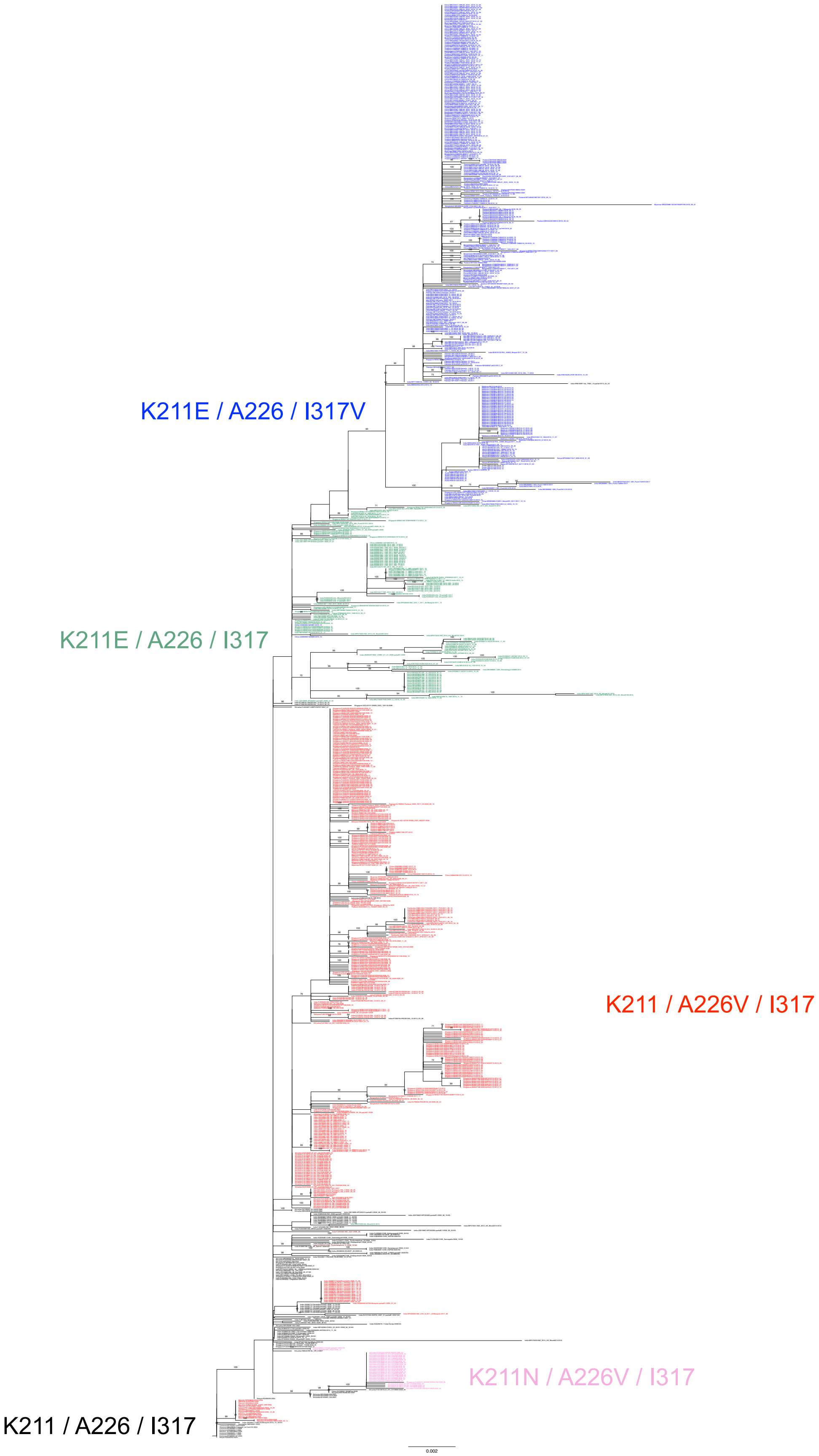

Figure S3. The Maximum Likelihood tree of 962 E1 sequences (1317 bp) of Indian Ocean Lineage (IOL) of CHIKV E1 constructed by IQ-TREE under TNe+G4. The combination of amino acid variations in E1 K211/A226/I317, K211N/A226V/I317, K211/A226V/I317, K211E/A226/I317, and K211E/A226/I317V correspond to the sequence color black, pink, red, green, and blue, respectively. The number adjacent to the branch indicates the support score of ultrafast bootstrap.
